# Association of pre-treatment sleep characteristics with reproductive outcomes among women undergoing assisted reproductive technology: a retrospective cohort study

**DOI:** 10.3389/fendo.2026.1965444

**Published:** 2026-10-30

**Authors:** Yating Li, Su Chen, Pengcheng Xu, Yangqin Peng, Jianxiang Fang, Zixiang Huang, Berthold Hocher, Junhua Zhou, Shujuan Ma

**Affiliations:** 1 School of Public Health, Hunan Normal University, Hunan, China; 2 Clinical Research Center for Reproduction and Genetics in Hunan, Reproductive and Genetic Hospital of CITIC-Xiangya, Hunan, China; 3 University Medical Centre Mannheim, University of Heidelberg, Mannheim, Germany

**Keywords:** cumulative live birth, embryo development, female, *in vitro* fertilization, sleep

## Abstract

**Objective:**

To investigate the associations between pre-treatment sleep characteristics and reproductive outcomes in infertile women undergoing assisted reproductive technology (ART) and to explore whether embryo development may represent a potential pathway linking sleep characteristics with cumulative live birth.

**Methods:**

In this retrospective cohort study, pre-treatment sleep characteristics before ovarian stimulation were assessed using the Pittsburgh Sleep Quality Index (PSQI). The primary outcome was cumulative live birth, while secondary outcomes included embryo developmental parameters. Associations with ovarian reserve indicators were also evaluated. Multivariable regression, restricted cubic spline, exploratory mediation, and sensitivity analyses were performed.

**Results:**

In an analysis of 11,090 included oocyte retrieval cycles, women with very poor sleep quality had a lower 1-year cumulative live birth rate (CLBR) (RR = 0.927, 95% CI: 0.881–0.977, P = 0.004). Sleep duration of 5 to less than 6 hours significantly reduced both 1-year (RR = 0.788, 95% CI: 0.737–0.843, P < 0.001) and 2-year CLBR (RR = 0.798, 95% CI: 0.748–0.852, P < 0.001). Furthermore, severe impairment in sleep efficiency, severe sleep disturbances, and severe daytime dysfunction were associated with lower CLBRs (P < 0.05). Secondary outcomes showed that poor sleep quality, short sleep duration of less than 5 hours, and use of sleeping medication 1–2 times per week correlated with lower available embryo rates (P < 0.05). Very bad subjective sleep quality was associated with an increased slow-cleaving embryo rate and high fragmentation rate (P < 0.05). Restricted cubic spline analyses suggested non-linear associations between sleep characteristics and ovarian reserve indicators. Exploratory mediation analyses suggested that embryo development may partially explain the associations of very bad subjective sleep quality and mild impairment in sleep latency with CLBRs.

**Conclusion:**

Poor sleep quality was associated with lower cumulative live birth outcomes and poorer cleavage-stage embryo development among women undergoing ART. Exploratory mediation analyses suggested that embryo development may partially explain the observed associations between sleep characteristics and CLBRs. As a potentially modifiable factor, sleep characteristics may represent an important consideration in optimizing reproductive care, although further prospective studies are needed.

## Introduction

Infertility is defined as the failure to achieve a clinical pregnancy after at least one year of unprotected sexual intercourse among couples of reproductive age (1). It is estimated that 8% to 12% of couples worldwide of childbearing age are affected by this condition (2). Relevant statistics indicate that approximately one quarter of couples attempting conception in China suffer from infertility (3). At present, assisted reproductive technology (ART) serves as the most effective therapeutic option for women who fail to conceive naturally. Nevertheless, ART is accompanied by high economic costs and limited efficacy. According to data from the European IVF Monitoring Consortium (4), the overall clinical pregnancy rate stood at 34% in 2018, implying that a large number of infertile patients still fail to achieve successful pregnancy. Accordingly, it is essential to explore the potential risk factors influencing ART outcomes.

As a core physiological process that maintains biological rhythms and regulates endocrine function, sleep is intrinsically linked to reproductive health (5, 6). Accumulating evidence has demonstrated that irregular sleep patterns may exert adverse impacts on female fertility through multiple biological pathways (7). Existing studies have confirmed that sleep disturbances interfere with hormonal rhythmicity via the hypothalamic-pituitary-gonadal axis and inhibit the secretion of sex hormones, thereby compromising female reproductive potential (5). Among infertile patients undergoing *in vitro* fertilization (IVF) treatment, sleep status is recognized as a prevalent factor closely associated with psychological stress; indeed, roughly 30% to 60% of women report impaired sleep quality before and after IVF cycles (8, 9). However, current research findings regarding the association between sleep conditions and ART outcomes remain inconsistent. A study conducted among Danish women receiving fertility treatment observed a non-significant upward trend in clinical pregnancy rates among participants with favorable sleep quality (10). In a prospective observational cohort study of 3,183 women, sleep duration was not significantly associated with any IVF outcomes (11). In contrast, another prospective cohort study involving 1,276 women undergoing IVF or intracytoplasmic sperm injection (ICSI) treatment found that women with a sleep duration of less than seven hours per night had fewer retrieved and mature oocytes compared with those sleeping 7 to 8 hours nightly, while those sleeping 9 to 10 hours per night presented lower chances of achieving clinical pregnancy (12). Such divergent results suggest that the relationship between sleep status and ART outcomes may be modulated by multiple confounding factors, including population characteristics, treatment protocols, follow-up endpoints, and sample size. Further large-sample and high-quality clinical investigations are therefore warranted to clarify this association.

Sleep constitutes a crucial and modifiable determinant of general health; hence, clarifying its influence on IVF/ICSI outcomes is of significant clinical importance. Although several studies have explored the correlation between sleep conditions and IVF/ICSI outcomes, substantial limitations remain in the current evidence base. Firstly, relevant conclusions are controversial and inconclusive. Most existing studies focus solely on the correlations between sleep indicators and clinical pregnancy or live birth rates (11), failing to elucidate their intrinsic associations with embryo development, which is a pivotal intermediate indicator. Secondly, a limited number of studies concerning sleep and embryo development are restricted by insufficient sample sizes, and few have analyzed the link between sleep status and the cumulative live birth rate(CLBR) following IVF/ICSI treatment (12, 13).

The present study aims to explore whether the sleep status of infertile women during the month prior to oocyte retrieval is correlated with cumulative live birth outcomes and cleavage-stage embryo development after IVF/ICSI treatment. In addition, the findings of this study will provide a solid foundation for future prospective research focusing on sleep intervention strategies to optimize IVF/ICSI clinical outcomes and facilitate the further development of assisted reproductive medicine.

## Methods

### Study design and study population

This was a retrospective cohort study based on the CXART database, a continuously updated and standardized clinical database for ART that covers the full cycle of care including preoperative surveys, diagnosis and treatment records, laboratory data, test results and pregnancy outcomes (14). Data were mainly obtained from the Survey sub-cohort of the CXART database, from which exposure, confounding and outcome data were all derived. And we enrolled women who underwent ART treatment at the Reproductive and Genetic Hospital of CITIC-Xiangya from October 2017 to August 2019 and completed the Pittsburgh Sleep Quality Index (PSQI) for sleep quality assessment. Exclusion criteria for analyzing the influence of sleep quality on ovarian reserve included invalid questionnaires, female age more than 45 years old, missing anti-Müllerian hormone (AMH) data, an interval of more than 6 months between AMH detection and PSQI investigation, and cycles failing quality control; we further excluded women who discontinued ART treatment, cycles with two or more oocyte retrievals at our hospital, oocyte donation cycles, natural cycles, *in vitro* maturation cycles, preimplantation genetic testing for monogenic disorders or structural rearrangements cycles, and cycles with an interval of more than 3 months between PSQI assessment and oocyte retrieval, included cycles with ≥ 3 inseminated oocytes for the analysis of cleavage-stage embryo development-related indicators, and finally excluded cycles with uterine malformation and preimplantation genetic testing for aneuploidy for the main analysis of the effect of sleep status on cumulative live birth (**Figure 1**). For the exploratory mediation analyses, the cohort was derived from the 11,300 cycles included in the analysis of cleavage-stage embryo development-related indicators. After further excluding cycles with uterine malformations and preimplantation genetic testing for aneuploidy, 10,176 cycles were included in the mediation analyses.

**Figure 1:**
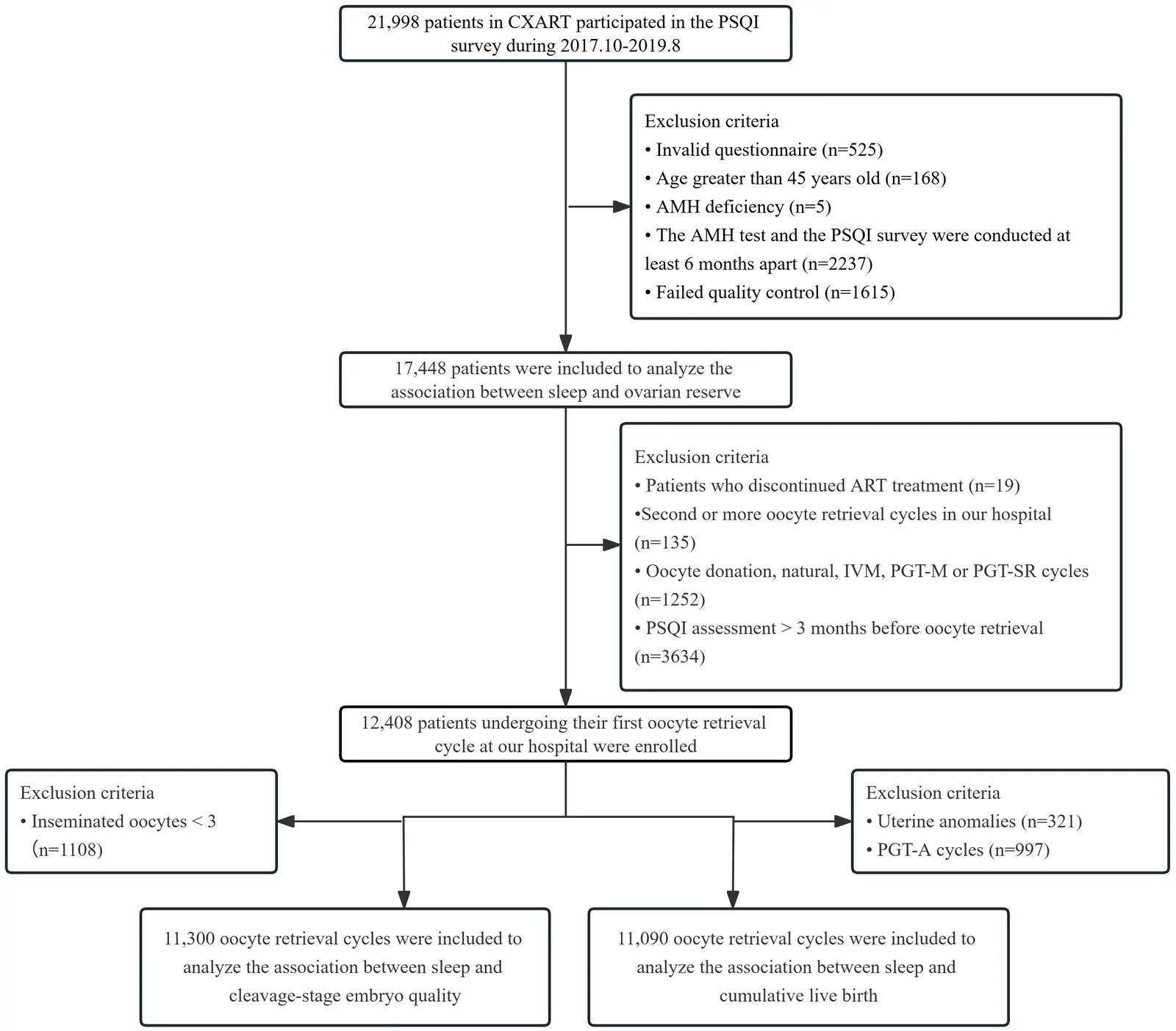
Flowchart of the participants through the study, detailing the inclusion and exclusion criteria. PSQI, pittsburgh sleep quality Index; AMH, anti-Müllerian hormone; ART, assisted reproductive technology; IVM, *in vitro* maturation; PGT-M, preimplantation genetic testing for monogenic disorders; PGT-SR, preimplantation genetic testing for structural rearrangements; PGT-A, preimplantation genetic testing for aneuploidy.

### Sleep characteristics assessment

Sleep quality was comprehensively evaluated using the PSQI. The PSQI was independently completed by participants on the day of enrollment in accordance with the provided instructions. To ensure data integrity, repeated core questions were designed as quality control items to assess response consistency and the completion quality of each scale. The PSQI reflects sleep status over the previous month and comprises 19 self-rated items (15). The PSQI assesses seven components: subjective sleep quality (very good, fairly good, fairly bad, very bad), sleep latency (0, 1–2, 3–4, 5–6 points), sleep duration (> 7 h, 6–7 h, 5–<6 h, < 5 h), habitual sleep efficiency (≥ 85%, 75% –< 85%, 65% –<75%, <65%), sleep disturbances (0, 1–9, 10–18, 19–27 points), use of sleeping medication (none, <1 time/week, 1–2 times/week, ≥3 times/week), and daytime dysfunction (0, 1–2, 3–4, 5–6 points). Each component score ranged from 0 to 3 points per standard scale criteria, with 0 indicating no difficulty and 3 indicating severe difficulty. Scores of 1 and 2 were defined as mild and moderate impairment, respectively. The global PSQI score ranges from 0 to 21, with higher scores indicating worse sleep quality. Based on global scores, sleep quality was classified into four levels: good (0–5), fair (6–10), poor (11–15), and very poor (16–21) (16).

### Assisted reproductive outcomes

The primary outcome was cumulative live birth. One-year and two-year cumulative live birth were defined as achieving at least one live birth following an embryo transfer performed within 12 or 24 months, respectively, from the date of oocyte retrieval. This included transfers of all fresh and subsequent frozen-thawed embryos obtained from that cycle (17). Secondary outcomes included ovarian reserve and cleavage-stage embryo development parameters. Ovarian reserve was assessed using AMH and antral follicle count (AFC). Cleavage-stage embryo development parameters included: available embryo rate (number of 2PN-fertilized embryos with cell count ≥ 6 and fragmentation grade I or II/number of normally cleaved embryos); slow-cleaving embryo rate (number of 2PN-fertilized embryos with cell count < 7/number of normally cleaved embryos); high-fragmentation embryo rate (number of 2PN-fertilized embryos with fragmentation grade III or IV/number of normally cleaved embryos). Normally cleaved embryos were defined as 2PN-fertilized embryos with a cell count ≥ 2. embryo development and cleavage stage were evaluated according to the Istanbul Consensus (18).

### Statistical analysis

Sleep characteristics were analyzed as both continuous and categorical variables. Continuous variables were expressed as medians (interquartile range, IQR), and categorical variables were expressed as frequencies (percentages). When treated as continuous variables (PSQI score, sleep duration, sleep latency, and habitual sleep efficiency), restricted cubic spline models were used to explore linear and non-linear associations between sleep status and ovarian reserve (AMH and AFC). Three knots were used, with knot locations corresponding to the 10th, 50th, and 90th percentiles of each continuous sleep characteristic. The reference value was determined based on the estimated inflection point from segmented regression. Model estimates were presented as differences in AMH or AFC relative to the reference value. For IVF/ICSI outcome analysis, sleep quality was divided into four groups according to global PSQI scores: good, fair, poor, and very poor. In addition, each PSQI component (subjective sleep quality, sleep latency, sleep duration, habitual sleep efficiency, sleep disturbances, use of sleeping medication, and daytime dysfunction) was analyzed as an independent explanatory variable. For the primary IVF/ICSI outcome (cumulative live birth), overlap-weighted quasi-Poisson regression with a log link was used to estimate adjusted risk ratios (RRs) and 95% confidence intervals (CIs), while accounting for potential baseline imbalances across sleep-characteristic groups. Cumulative live birth was defined as a binary outcome (yes/no). Variance estimates were obtained using the dispersion parameter of the quasi-Poisson model. For secondary outcomes regarding cleavage-stage embryo development, beta-binomial or zero-inflated beta-binomial regression models were used, considering the proportional distribution of the outcomes (between 0 and 1), non-normality, and potential zero inflation. When both models converged successfully, the zero-inflation component was assessed based on the statistical significance of its intercept. The zero-inflated beta-binomial model was selected when the intercept was statistically significant (P < 0.05); otherwise, the standard beta-binomial model was used. Adjusted odds ratios (ORs) with 95% CIs were calculated. Multivariable statistical analyses were adjusted for relevant confounders based on previous literature (19–21). Because the relevant covariates differed according to the outcome and analytical model, adjustment sets were specified separately for each analysis. The specific covariates included in each model are detailed in the corresponding table notes and figure legends. In addition, participants with disqualified questionnaire quality had been excluded during the inclusion and exclusion stage.

Based on the counterfactual potential outcome framework (22), exploratory mediation analyses were further performed to investigate whether embryo development may represent a potential pathway linking sleep characteristics with cumulative live birth. beta-binomial/zero-inflated beta-binomial regression and overlap-weighted quasi-Poisson regression were used for the mediation model and outcome model, respectively, to ensure consistency with the model framework of the main effect analysis. The bootstrap resampling method (1,000 iterations) was used to estimate the average indirect effect, average direct effect, and total effect, as well as the proportion mediated. Effects were presented as adjusted RRs and 95% CIs.

To verify the reliability of the results, sensitivity analyses were additionally conducted by re-including participants excluded for failing quality control items, and 1-year and 2-year cumulative live births were analyzed using the full dataset to further confirm the stability of the conclusions. Second, to assess the robustness of the variance estimation for the binary cumulative live birth outcome, overlap-weighted modified Poisson regression with HC0 robust sandwich variance estimation was performed. All analyses were performed using R software (version 4.5.2), and a p-value < 0.05 was considered statistically significant.

## Results

### Baseline characteristics of all participants

A total of 11,090 oocyte retrieval cycles were included in the analysis of cumulative live birth. The median interval between PSQI assessment and oocyte retrieval was 66 days (IQR, 49.25–81 days). Participants were stratified into four groups based on PSQI scores: good (n = 7,885), fair (n = 2,704), poor (n = 469), and very poor sleep quality (n = 32). Detailed demographic and clinical characteristics are summarized in **Table 1**. Median age, BMI and age at menarche were 31.0 years, 21.5 kg/m² and 13.0 years, respectively. Most participants were never smokers (93.4%), while current smokers accounted for 1.3%. Secondary infertility was predominant (58.9%), and 82.4% had no polycystic ovary syndrome. Additionally, 11,300 oocyte retrieval cycles were included in the analysis of the secondary outcomes (cleavage-stage embryo development), with characteristics stratified by sleep quality presented in **Supplementary Table 1**.

**Table 1:** Demographic and baseline clinical characteristics by sleep quality among participants in a cumulative live birth cohort.

Characteristics	Total (N=11090)	Good group (N=7885)	Fair group (N=2704)	Poor group (N=469)	Very poor group (N=32)	*P* value
Age, years, median (IQR)	31.0 (28.0, 34.0)	31.0 (28.0, 35.0)	30.0 (28.0, 34.0)	31.0 (28.0, 35.0)	32.0 (29.0, 35.0)	0.006
BMI, kg/m2, median (IQR)	21.5 (19.8, 23.1)	21.5 (19.9, 23.2)	21.2 (19.6, 22.9)	21.1 (19.6, 22.8)	20.8 (19.0, 22.4)	<0.001
Menarche age, median (IQR)	13.0 (12.0, 14.0)	13.0 (13.0, 14.0)	13.0 (12.0, 14.0)	13.0 (13.0, 14.0)	13.0 (12.0, 14.0)	0.062
Smoking status						0.039
Never smoked	10,354 (93.4%)	7399 (93.9%)	2500 (92.5%)	427 (91.0%)	28 (87.5%)	
Ever smoked, now quit	588 (5.3%)	393 (5.0%)	160 (5.9%)	32 (6.8%)	3 (9.4%)	
Current smoker	145 (1.3%)	91 (1.2%)	43 (1.6%)	10 (2.1%)	1 (3.1%)	
AMH, ng/ml, median (IQR)	4.0 (2.0, 7.2)	4.0 (2.0, 7.3)	4.1 (2.1, 7.1)	3.8 (1.9, 6.8)	3.3 (1.7, 6.3)	0.483
FSH, mIU/mL, median (IQR)	5.8 (5.0, 6.9)	5.8 (5.0, 6.9)	5.9 (5.0, 6.9)	5.8 (4.9, 6.9)	5.9 (4.9, 6.9)	0.603
AFC, median (IQR)	18.0 (11.0, 30.0)	18.0 (11.0, 30.0)	18.0 (11.0, 29.0)	17.0 (10.0, 28.0)	13.0 (9.0, 28.0)	0.031
ART type						0.151
IVF	7,940 (71.6%)	5,651 (71.7%)	1,908 (70.6%)	359 (76.5%)	22 (68.8%)	
ICSI	2,376 (21.4%)	1,694 (21.5%)	596 (22.0%)	80 (17.1%)	6 (18.8%)	
IVF+ICSI	774 (7.0%)	540 (6.8%)	200 (7.4%)	30 (6.4%)	4 (12.5%)	
Ovarian stimulation protocol						0.148
GnRH agonist	8,402 (75.8%)	5,998 (76.1%)	2,049 (75.8%)	331 (70.6%)	24 (75.0%)	
GnRH antagonist	1,705 (15.4%)	1,211 (15.4%)	406 (15.0%)	83 (17.7%)	5 (15.6%)	
Others	983 (8.9%)	676 (8.6%)	249 (9.2%)	55 (11.7%)	3 (9.4%)	
Infertility type						0.419
Primary infertility	4,555 (41.1%)	3202 (40.6%)	1140 (42.2%)	201 (42.9%)	12 (37.5%)	
Secondary infertility	6,535 (58.9%)	4683 (59.4%)	1564 (57.8%)	268 (57.1%)	20 (62.5%)	
Infertility related diseases						
Severe OAT						0.477
No	9,666 (87.2%)	6,864 (87.1%)	2,348 (86.8%)	425 (90.6%)	29 (90.6%)	
Yes	1,424 (12.8%)	1,021 (12.9%)	356 (13.2%)	44 (9.4%)	3 (9.4%)	
Donor sperm						0.116
No	10,463 (94.3%)	7,429 (94.2%)	2,567 (94.9%)	437 (93.2%)	30 (93.8%)	
Yes	627 (5.7%)	456 (5.8%)	137 (5.1%)	32 (6.8%)	2 (6.3%)	
PCOS						0.341
No	9,136 (82.4%)	6484 (82.2%)	2229 (82.4%)	394 (84.0%)	29 (90.6%)	
Yes	1,954 (17.6%)	1401 (17.8%)	475 (17.6%)	75 (16.0%)	3 (9.4%)	
IUA						0.008
No	10,587 (95.5%)	7,518 (95.3%)	2,599 (96.1%)	443 (94.5%)	27 (84.4%)	
Yes	503 (4.5%)	367 (4.7%)	105 (3.9%)	26 (5.5%)	5 (15.6%)	
RIF						0.429
No	10,846 (97.8%)	7,723 (97.9%)	2,635 (97.4%)	457 (97.4%)	31 (96.9%)	
Yes	244 (2.2%)	162 (2.1%)	69 (2.6%)	12 (2.6%)	1 (3.1%)	
Recurrent miscarriage						0.238
No	10,858 (97.9%)	7,733 (98.1%)	2,635 (97.4%)	459 (97.9%)	31 (96.9%)	
Yes	232 (2.1%)	152 (1.9%)	69 (2.6%)	10 (2.1%)	1 (3.1%)	
Untreated hydrosalpinx						0.314
No	10,233 (92.3%)	7,282 (92.4%)	2,488 (92.0%)	436 (93.0%)	27 (84.4%)	
Yes	857 (7.7%)	603 (7.6%)	216 (8.0%)	33 (7.0%)	5 (15.6%)	
Cumulative live birth (1-year)						0.206
NO	4,921 (44.4%)	3500 (44.4%)	1177 (43.5%)	228 (48.6%)	16 (50.0%)	
Yes	6,169 (55.6%)	4385 (55.6%)	1527 (56.5%)	241 (51.4%)	16 (50.0%)	
Cumulative live birth (2-year)						0.277
NO	4719(42.6%)	3355 (42.5%)	1131 (41.8%)	218 (46.5%)	15 (46.9%)	
Yes	6371(57.4%)	4530 (57.5%)	1573 (58.2%)	251 (53.5%)	17 (53.1%)	

Participants were divided into four groups according to the Pittsburgh Sleep Quality Index (PSQI) score (range: 0–21): good group (0–5), fair group (6–10), poor group (11–15), and very poor group (16–21). Slight missing data existed in individual variables. All analyses were performed using valid observed data.

IQR, interquartile range; BMI, body mass index; AMH, anti-Müllerian hormone; FSH, follicle stimulating hormone; AFC, antral follicle count; ART, assisted reproductive technology; IVF, in vitro fertilization; ICSI, intracytoplasmic sperm injection; GnRH, gonadotropin-releasing hormone; OAT, oligoasthenoteratozoospermia; PCOS, polycystic ovary syndrome; IUA, moderate to severe intrauterine adhesions; RIF, recurrent implantation failure.

In the correlation analysis between sleep and ovarian reserve, after adjusting for female age, BMI, and smoking status, significant overall associations were observed between sleep duration and levels of AMH and AFC (*P* = 0.011 and *P* = 0.007, respectively). Both associations exhibited non-linear relationships (*P*_non-linear_ = 0.009 and *P*_non-linear_ = 0.002, respectively). Restricted cubic spline curves revealed that AMH and AFC levels initially increased and subsequently decreased with prolonged sleep duration, indicating an inverted U-shaped dose–response relationship (**Supplementary Figures 1**, **2**).

### Association of sleep characteristics with cumulative live birth outcomes

After stratification by sleep quality, the 1-year CLBR were 55.6% (4385/7885), 56.5% (1527/2704), 51.4% (241/469) and 50.0% (16/32) for good, fair, poor and very poor sleep quality groups, while the corresponding 2-year rates stood at 57.5%, 58.2%, 53.5% and 53.1% respectively (**Table 1**). The relationships between sleep characteristics and cumulative live birth are shown in **Figure 2**. Sleep quality, sleep duration, habitual sleep efficiency, sleep disturbances, and daytime dysfunction were associated with the 1-year CLBR. Women with very poor sleep quality had a lower 1-year CLBR (RR = 0.927, 95% CI: 0.881–0.977, P = 0.004). Women sleeping 5 to less than 6 hours or less than 5 hours had a lower 1-year CLBR than those sleeping more than 7 hours (RR = 0.788, 95% CI: 0.737–0.843, P < 0.001; RR = 0.871, 95% CI: 0.815–0.931, *P* < 0.001). Women with a sleep efficiency of less than 65% had a lower 1-year CLBR than those with 85% or higher (RR = 0.904, 95% CI: 0.859–0.951, *P* < 0.001). Furthermore, severe sleep disturbances and daytime dysfunction correlated with a lower 1-year CLBR (RR = 0.934, 95% CI: 0.892–0.979, *P* = 0.004; RR = 0.800, 95% CI: 0.757–0.845, *P* < 0.001, respectively). At the 2-year follow-up, the overall pattern was broadly consistent with that observed for 1-year CLBR. Compared with the reference groups, sleep duration of 5 to less than 6 hours, sleep efficiency of less than 65%, moderate-to-severe sleep disturbances, and severe daytime dysfunction remained significantly associated with lower 2-year CLBRs.

**Figure 2:**
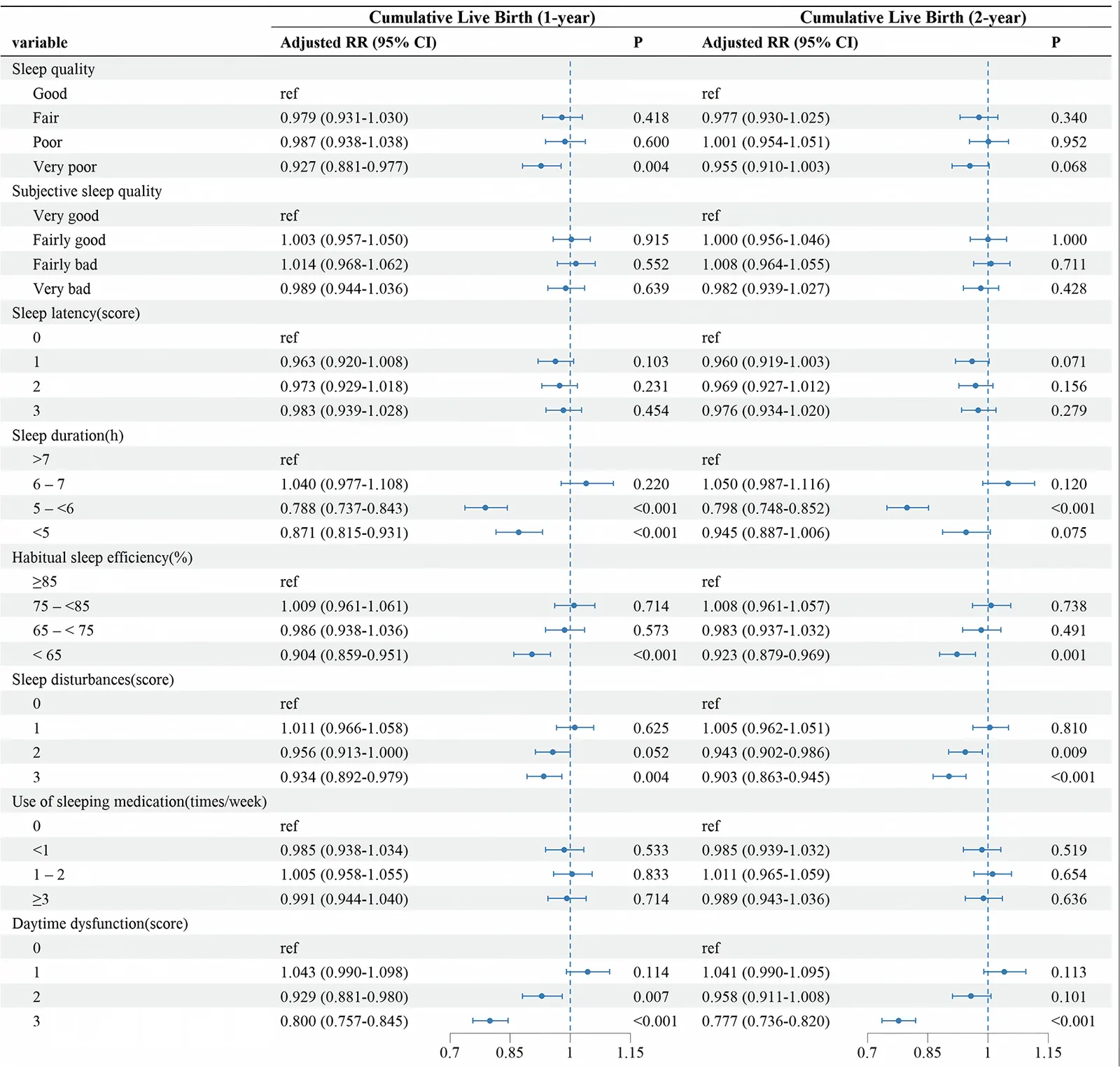
Forest plot of the relationship between sleep characteristics and cumulative live birth rate in 11,090 oocyte retrieval cycles, displaying the outcomes of overlapping weighted quasi-Poisson regression analyses across levels of individual PSQI items, exploring the association between sleep characteristics and cumulative live birth rate. Adjusted factors included female age, body mass index, type of infertility, polycystic ovary syndrome, severe oligoasthenoteratozoospermia, moderate to severe intrauterine adhesions, recurrent implantation failure, recurrent miscarriage, untreated hydrosalpinx, ovarian stimulation protocol, and assisted reproductive technology type.

In sensitivity analyses, participants initially excluded for failing quality control were re-enrolled to verify findings with an expanded sample size. Overall, the results were generally consistent with those of the main analysis for most sleep characteristics and reproductive outcomes. However, the association between severe sleep disturbances and one-year cumulative live birth showed a reversal in direction, with an RR of 1.153 in the sensitivity analysis compared with 0.934 in the main analysis. Detailed results are presented in **Supplementary Table 2**.

In an additional sensitivity analysis using overlap-weighted modified Poisson regression with HC0 robust sandwich variance estimation, the point estimates were consistent with those from the primary analysis. For the very poor sleep group, the RR for 1-year cumulative live birth remained 0.927. Detailed results are presented in **Supplementary Figure 3**.

### Association of sleep characteristics with cleavage-stage embryo development outcomes

Results for the association between sleep characteristics and cleavage-stage embryo development are presented in **Table 2**. Participants with poor sleep quality had a lower available embryo rate than those with good sleep quality (OR = 0.875, 95% CI: 0.783–0.979, *P* = 0.019). Participants with fairly good subjective sleep quality had a lower available embryo rate than those with very good subjective sleep quality (OR = 0.943, 95% CI: 0.894–0.995, *P* = 0.032). Furthermore, participants with very bad subjective sleep quality had a higher slow-cleaving embryo rate and a higher high fragmentation rate (OR = 1.154, 95% CI: 1.035–1.288, *P* = 0.010; OR = 1.173, 95% CI: 1.030–1.337, *P* = 0.016).

**Table 2:** Association of sleep characteristics with cleavage-stage embryo development in 11,300 oocyte retrieval cycles.

Sleep characteristics	Available embryo rate, %		Slow-cleaving embryo rate, %		High fragmentation rate, %	
	Adjusted OR (95% CI)	P	Adjusted OR (95% CI)	P	Adjusted OR (95% CI)	P
Sleep quality						
Good	ref		ref		ref	
Fair	1.002 (0.950, 1.057)	0.936	1.008 (0.953, 1.067)	0.778	1.053 (0.981, 1.130)	0.153
Poor	**0.875 (0.783, 0.979)**	0.019*	1.068 (0.944, 1.209)	0.294	0.982 (0.841, 1.146)	0.816
Very poor	0.952 (0.629, 1.443)	0.818	1.086 (0.699, 1.689)	0.713	0.940 (0.540, 1.637)	0.827
Subjective sleep quality						
Very good	ref		ref		ref	
Fairly good	**0.943(0.894, 0.995)**	0.032*	0.994 (0.939, 1.052)	0.835	0.991 (0.922, 1.064)	0.796
Fairly bad	0.980 (0.916, 1.049)	0.568	0.980 (0.912, 1.054)	0.592	0.986 (0.901, 1.080)	0.764
Very bad	0.948 (0.857, 1.048)	0.295	**1.154 (1.035, 1.288)**	0.010*	**1.173 (1.030, 1.337)**	0.016*
Sleep latency (score)						
0	ref		ref		ref	
1	1.001 (0.949, 1.056)	0.966	1.050 (0.992, 1.112)	0.091	1.073 (0.999, 1.152)	0.054
2	0.992 (0.926, 1.062)	0.812	1.051 (0.977, 1.132)	0.184	1.080 (0.985, 1.184)	0.101
3	0.955 (0.833, 1.096)	0.514	1.074 (0.925, 1.246)	0.349	1.056 (0.877, 1.272)	0.566
Sleep duration (h)						
>7	ref		ref		ref	
6 – 7	0.973 (0.927, 1.021)	0.269	0.962 (0.913, 1.013)	0.139	0.977 (0.916, 1.042)	0.480
5 – <6	0.859 (0.667, 1.105)	0.236	1.090 (0.832, 1.427)	0.532	1.153 (0.825, 1.612)	0.403
<5	**0.647(0.326, 0.985)**	0.044*	0.662 (0.379, 1.158)	0.148	0.519 (0.222, 1.213)	0.130
Habitual sleep efficiency (%)						
≥85	ref		ref		ref	
75 – <85	1.009 (0.955, 1.067)	0.743	1.004 (0.946, 1.065)	0.902	1.009 (0.937, 1.085)	0.820
65 – < 75	0.978 (0.881, 1.086)	0.682	1.022 (0.913, 1.143)	0.707	0.906 (0.786, 1.046)	0.177
< 65	0.911 (0.765, 1.086)	0.298	0.935 (0.772, 1.132)	0.489	0.912 (0.712, 1.169)	0.469
Sleep disturbances (score)						
0	ref		ref		ref	
1	0.997 (0.949, 1.048)	0.905	0.982 (0.931, 1.035)	0.493	1.038 (0.972, 1.110)	0.267
2	0.954 (0.843, 1.079)	0.452	1.092 (0.956, 1.247)	0.195	1.037 (0.878, 1.224)	0.671
3	1.569 (0.724, 3.399)	0.253	1.730 (0.770, 3.883)	0.184	1.741 (0.764, 3.969)	0.187
Use of sleeping medication (times/week)						
0	ref		ref		ref	
<1	0.997 (0.942, 1.054)	0.908	0.967 (0.911, 1.027)	0.279	1.036 (0.962, 1.116)	0.344
1 – 2	**0.928(0.863, 0.998)**	0.043*	0.928 (0.858, 1.003)	0.059	0.910 (0.824, 1.005)	0.063
≥3	0.990 (0.885, 1.108)	0.863	1.077 (0.955, 1.215)	0.228	1.066 (0.920, 1.235)	0.397
Daytime dysfunction (score)						
0	ref		ref		ref	
1	0.988 (0.939, 1.040)	0.649	1.003 (0.950, 1.059)	0.913	0.973 (0.909, 1.041)	0.425
2	0.950 (0.867, 1.041)	0.271	1.033 (0.936, 1.141)	0.517	1.025 (0.907, 1.159)	0.692
3	0.762 (0.491, 1.181)	0.224	1.337 (0.832, 2.151)	0.231	1.339 (0.777, 2.307)	0.293

Data are presented as odds ratios (OR) with 95% confidence intervals (CI). Adjusted factors included female age, body mass index, type of infertility, polycystic ovary syndrome, severe oligoasthenoteratozoospermia, ovarian stimulation protocol, and insemination method. The available embryo rate was analyzed using zero-inflated beta-binomial regression, whereas the slow-cleaving embryo rate and high-fragmentation embryo rate were analyzed using beta-binomial regression.

Bold values indicate statistically significant differences (P < 0.05).

Those with a sleep duration of less than 5 hours had a lower available embryo rate than participants with a sleep duration of more than 7 hours (OR = 0.647, 95% CI: 0.326–0.985, *P* = 0.044). In addition, use of sleeping medication 1–2 times per week was associated with a significantly lower available embryo rate than no use of sleeping medication (OR = 0.928, 95% CI: 0.863–0.998, *P* = 0.043).

### Exploratory analysis of potential pathways linking sleep characteristics, cleavage-stage embryo development, and cumulative live birth

A total of 144 exploratory mediation analyses were performed. Exploratory mediation analyses suggested that the slow-cleaving embryo rate and high fragmentation rate may partially explain the association between very bad subjective sleep quality and cumulative live birth rates (**Figure 3**). Specifically, nominally significant indirect effects were observed for both 1-year and 2-year cumulative live birth rates (1-year: RR = 0.981, 95% CI: 0.964–0.998, *P* = 0.020; 2-year: RR = 0.980, 95% CI: 0.963–0.998, *P* = 0.022 for slow-cleaving embryo rate; and 1-year: RR = 0.985, 95% CI: 0.972–0.998, *P* = 0.024; 2-year: RR = 0.985, 95% CI: 0.971–0.998, *P* = 0.032 for high fragmentation rate). Additionally, mild impairment in sleep latency showed a nominally significant potential indirect association with cumulative live birth through the high fragmentation rate (1-year: RR = 0.994, 95% CI: 0.988–1.000, *P* = 0.044; 2-year: RR = 0.994, 95% CI: 0.987–1.000, *P* = 0.048). No other indirect effects reached nominal statistical significance. Given the multiple comparisons, these findings should be interpreted as exploratory and hypothesis-generating rather than confirmatory. Detailed results are provided in **Supplementary Tables 3–5**.

**Figure 3:**
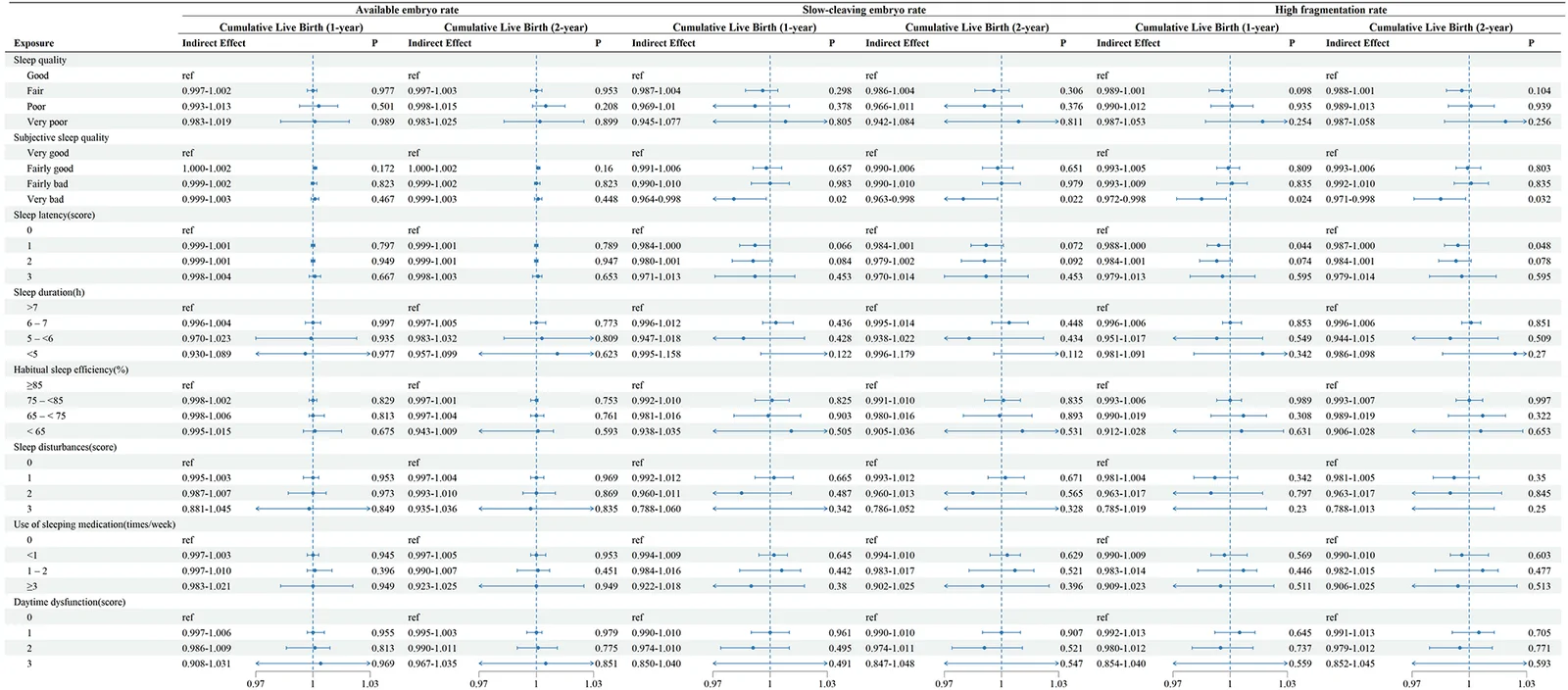
Forest plot showing exploratory mediation analyses involving three indicators of cleavage-stage embryo development in the associations between sleep characteristics and cumulative live birth. IRR represents incidence rate ratio. All effect estimates are presented with 95% confidence intervals. Adjusted confounding factors included female age, body mass index, type of infertility, polycystic ovary syndrome, severe oligoasthenoteratozoospermia, moderate to severe intrauterine adhesions, recurrent implantation failure, recurrent miscarriage, untreated hydrosalpinx, ovarian stimulation protocol, and assisted reproductive technology type.

## Discussion

The results revealed that sleep quality, sleep duration, habitual sleep efficiency, sleep disturbances, and daytime dysfunction were all associated with cumulative live birth in a single oocyte retrieval cycle. The present study also found that women with poor sleep quality had a lower available embryo rate, while those with very bad subjective sleep quality had a higher slow-cleaving embryo rate and high fragmentation rate. In addition, nonlinear associations were found between sleep duration and AMH as well as AFC.

Our data showed an inverse association between very poor sleep quality and 1-year cumulative live birth. A prospective cohort study using the PSQI scale found that sleep quality was associated with live birth (11), which is consistent with our findings. Another study reached a similar conclusion that women with higher PSQI scores had significantly lower odds of successful conception after IVF/ICSI (23). Nevertheless, no significant association was found between sleep quality and 2-year cumulative live birth in this study. Given the small number of cycles in the very poor sleep quality group (n = 32), the statistical power to detect an association may have been limited. In addition, sleep duration was inverse association with 1-year and 2-year CLBR, although current findings from relevant studies remain inconsistent. A prospective cohort study using the PSQI scale found no association between sleep duration and IVF/ICSI live birth among 3,183 Chinese women (11) and a similar result was reported in another study of 365 women undergoing IVF/ICSI, in which sleep duration was not related to clinical pregnancy (24). This discrepancy may be attributed to differences in study design (retrospective vs. prospective) or the use of a single transfer as the study endpoint. Furthermore, Liu et al. included both fresh and frozen embryo transfers with subgroup analyses (11), whereas our study used cumulative live birth as the outcome.

Notably, we observed that habitual sleep efficiency, sleep disturbances, and daytime dysfunction were all positively associated with 1-year and 2-year CLBR. A previous study showed that the clinical pregnancy rate was significantly lower in patients with sleep efficiency < 85% than in those with sleep efficiency > 85%, consistent with our results (25). Studies on sleep disturbances and CLBR remain limited and have not reached consistent conclusions. Furthermore, few studies have identified an association between daytime dysfunction and cumulative live birth. The present study, based on a large sample, confirmed a significant correlation, which may be explained by the small sample sizes and insufficient statistical power in previous studies that failed to detect this association.

Using restricted cubic spline models, this study identified a non-linear association between sleep duration and levels of AMH as well as AFC, rather than a simple linear relationship. These findings indicate that both excessively short and long sleep duration may correlate with diminished ovarian reserve. It suggests that sleep duration exerts a non-unidirectional effect on ovarian function, and their interaction is complex. Mechanistically, insufficient sleep disrupts circadian rhythms and thereby inhibits melatonin secretion (26, 27). As a key endogenous antioxidant, melatonin modulates circadian rhythms, scavenges free radicals and alleviates oxidative stress (28, 29). Reduced melatonin impairs the local antioxidant defense system in the ovary and elevates oxidative stress, which damages oocytes, granulosa cells and the follicular microenvironment, and ultimately leads to decreased ovarian reserve (29, 30). The clinical implications and underlying mechanisms of prolonged sleep duration remain unclear and require further investigation.

We also observed that poor sleep quality and fairly good subjective sleep quality were associated with lower available embryo rates, while very bad subjective sleep quality was associated with higher slow-cleaving and high-fragmentation embryo rates. The results suggest that apart from overall sleep status, patients’ subjective perception of sleep may also be closely related to embryonic developmental outcomes. Furthermore, shorter sleep duration was linked to a lower available embryo rate, implying that sleep duration may also be involved in the regulation of embryonic development. We also observed that the use of sleeping medication 1–2 times per week was associated with a lower available embryo rate, but not with cumulative live birth. This discrepancy may indicate that early embryo-development outcomes do not necessarily translate into differences in cumulative live birth.

The potential mechanisms may include hypothalamic-pituitary-adrenal (HPA) axis dysfunction, inflammatory responses, oxidative stress and changes in reproductive hormone levels. Previous studies have confirmed that poor sleep quality can alter the secretion of inflammatory cytokines (31) and enhance HPA axis stress responses (32). Basal HPA axis activity is believed to continuously regulate peripheral inflammation (33). In turn, activated inflammation is closely correlated with oxidative stress, and their combined effects may further damage embryonic development (34). In addition, existing evidence has shown that sleep duration has associations with core reproductive hormones such as estradiol, luteal-phase progesterone and follicle-stimulating hormone (35, 36). Although the present study focused on short sleep duration in the general population rather than experimental sleep deprivation, relevant animal studies have demonstrated that sleep deprivation downregulates estrogen and follicle-stimulating hormone levels, elevates intracellular reactive oxygen species in oocytes, and ultimately hinders embryonic development (37). It is thus hypothesized that short sleep duration may trigger similar physiological perturbations, which is in line with the finding of impaired embryo quality in this study.

On this basis, exploratory mediation analyses were further conducted to investigate potential pathways linking sleep characteristics, cleavage-stage embryo development, and subsequent cumulative live birth. A total of 144 mediation models were evaluated, and six indirect associations reached nominal statistical significance. These findings should be interpreted as exploratory and hypothesis-generating given the multiple comparisons and lack of adjustment for multiple testing. Furthermore, mediated proportions exceeding 100% should be interpreted cautiously because this ratio-based measure may become unstable when the total effect is close to the null.

The associations between severe sleep disturbances and CLBR changed direction in the sensitivity analysis, with RRs changing from 0.934 to 1.153 (1-year) and from 0.903 to 1.120 (2-year). These discrepancies may reflect differences in the expanded analytic population, and these findings should therefore be interpreted cautiously.The observed associations between sleep duration and ART outcomes may provide insights for future sleep-related research in infertile populations. Unlike irreversible factors such as age and ovarian reserve, sleep duration is relatively easy to assess and potentially modifiable. Clinicians can quickly identify sleep problems via simple questionnaires, while the potential benefits of targeted interventions before embryo transfer for IVF/ICSI outcomes and CLBRs warrant further investigation. It is recommended to incorporate sleep assessment into the routine diagnosis and treatment of infertile patients and strengthen full-cycle sleep management. This study provides new insights into the relationship between sleep characteristics and assisted reproductive outcomes, highlighting potential pathways involving embryo development and supporting further research into sleep-related interventions in ART.

The main strengths of this study include its large sample size, comprehensive evaluation of sleep characteristics, and use of cumulative live birth as the primary outcome. The large sample size and adjustment for measured confounding factors such as age and BMI strengthened the robustness of the analyses. Independent analyses of individual PSQI components also allowed a more detailed assessment of their associations with IVF/ICSI outcomes. This study also has several limitations. First, the use of a subjective sleep assessment may have introduced information bias; future studies incorporating objective sleep measures may provide complementary evidence. Second, as a retrospective study, residual confounding from unmeasured or unavailable factors cannot be completely excluded. In particular, information on psychological distress, shift work, previous fertility treatment history, and some clinical and laboratory factors related to embryo culture and transfer was unavailable for adjustment. In addition, the database did not provide sufficient information to distinguish between different types or classes of sleeping medications. Third, the very poor sleep quality group was relatively small (n = 32), which may have limited the statistical power and precision of the estimates. Finally, this study focused on female sleep; future studies should consider the sleep characteristics of both partners.

## Conclusions

This study found that female sleep characteristics were associated with ART outcomes. Very poor sleep quality may be associated with lower 1-year CLBR. Patients with sleep duration 5 to less than 6 hours, sleep efficiency of less than 65%, severe sleep disturbances and daytime dysfunction exhibited significantly reduced 1-year and 2-year CLBRs. For the secondary outcomes of cleavage-stage embryo development, poor sleep quality, fairly good subjective sleep quality, sleep duration of less than 5 hours, and use of sleeping medication 1–2 times per week were all related to a lower available embryo rate. Meanwhile, very poor subjective sleep quality was associated with higher slow-cleaving embryo rate and higher fragmentation rate. Furthermore, exploratory mediation analyses suggested that cleavage-stage embryo development may represent a potential pathway linking sleep characteristics with CLBRs. These findings indicate that sleep disturbances are associated not only with reduced CLBRs but also with poorer early embryonic development. Sleep status represents a potentially modifiable factor in ART treatment. Sleep assessment and targeted interventions before embryo transfer may warrant further investigation as strategies to improve reproductive outcomes.

## Data Availability

The data analyzed in this study is subject to the following licenses/restrictions: The datasets used and analyzed during the current study are available from the corresponding author upon reasonable request. Requests to access these datasets should be directed to 790039940@qq.com.
