# Supplementary material for: Association of pre-treatment sleep characteristics with reproductive outcomes among women undergoing assisted reproductive technology: a retrospective cohort study
Source: Front Endocrinol (Lausanne). 2026 Oct 30;17:1965444. doi: 10.3389/fendo.2026.1965444 (PMC13759281; doi:10.3389/fendo.2026.1965444)
Supplement: Supplementary file 1 [file Supplementaryfile1.docx]

**Supplemental materials**

[Supplementary figure 1.	2](#_Toc5977)

[Supplementary figure 2.	3](#_Toc19874)

Supplementary figure 3............................................................................................................................................................................ 4

[Supplemental table 1.	5](#_Toc8955)

[Supplemental table 2.	6](#_Toc1288)

[Supplemental table 3.	7](#_Toc6283)

[Supplemental table 4.	9](#_Toc20786)

[Supplemental table 5.	11](#_Toc11547)

Supplementary figure 1. Restricted cubic spline models analyses were performed to explore the associations between sleep characteristics and ovarian reserve markers.The figure illustrates the associations between PSQI total score and AMH (Panel A), sleep latency and AMH (Panel B), sleep duration and AMH (Panel C), habitual sleep efficiency and AMH (Panel D). Adjustment factors included age, body mass index, and smoking status. Solid lines represent estimated effects, and shaded areas represent 95% confidence intervals. The horizontal dashed line at zero represents the reference level, and the vertical line indicates the reference value of the corresponding sleep characteristic. The y-axis represents the estimated difference in AMH relative to the reference value; negative values indicate lower estimated AMH than the reference value and do not represent negative absolute AMH concentrations. Abbreviations: PSQI=pittsburgh sleep quality index; AMH=anti-Müllerian hormone.

Supplementary figure 2. Restricted cubic spline models analyses were performed to explore the associations between sleep characteristics and ovarian reserve markers.The figure illustrates the associations between PSQI total score and AFC (Panel A), sleep latency and AFC (Panel B), sleep duration and AFC (Panel C), habitual sleep efficiency and AFC (Panel D). Adjustment factors included age, body mass index, and smoking status. Solid lines represent estimated effects, and shaded areas represent 95% confidence intervals. The horizontal dashed line at zero represents the reference level, and the vertical line indicates the reference value of the corresponding sleep characteristic. The y-axis represents the estimated difference in AFC relative to the reference value; negative values indicate lower estimated AFC than the reference value and do not represent negative absolute AFC counts. Abbreviations: PSQI=pittsburgh sleep quality index; AFC=antral follicle count.

Supplementary Figure 3. Forest plot of the sensitivity analysis of the relationship between sleep characteristics and cumulative live birth rate in 11,090 oocyte retrieval cycles, displaying the results of overlap-weighted modified Poisson regression with robust variance estimation for the overall PSQI sleep quality measure and individual PSQI dimensions. Adjusted factors included female age, body mass index, type of infertility, polycystic ovary syndrome, severe oligoasthenoteratozoospermia, moderate to severe intrauterine adhesions, recurrent implantation failure, recurrent miscarriage, untreated hydrosalpinx, ovarian stimulation protocol, and assisted reproductive technology type.

Supplemental table 1. **Demographic and baseline clinical characteristics by sleep quality among participants in a cleavage-stage embryo development cohort.**

|  | **Total**  **(N=11300)** | **Good group**  **(N=8043)** | **Fair group**  **(N=2751)** | **Poor group**  **(N=470)** | **Very poor group**  **(N=36)** | ***P* value** |
| --- | --- | --- | --- | --- | --- | --- |
| Age, years, median (IQR) | 31.0 (28.0, 34.0) | 31.0 (28.0, 35.0) | 30.0 (28.0, 34.0) | 31.0 (28.0, 35.0) | 33.0 (29.0, 35.0) | 0.002 |
| BMI, kg/m2, median (IQR) | 21.4 (19.8, 23.1) | 21.5 (19.9, 23.2) | 21.2 (19.6, 22.9) | 21.1 (19.6, 22.9) | 21.2 (19.1, 23.0) | <0.001 |
| Menarche age, median (IQR) | 13.0 (12.0, 14.0) | 13.0 (12.0, 14.0) | 13.0 (12.0, 14.0) | 13.0 (13.0, 14.0) | 13.0 (12.0, 14.0) | 0.055 |
| Smoking status |  |  |  |  |  | 0.318 |
| Never smoked | 10,544 (93.3%) | 7,528 (93.6%) | 2,553 (92.9%) | 430 (91.5%) | 33 (91.7%) |  |
| Ever smoked, now quit | 602 (5.3%) | 415 (5.2%) | 154 (5.6%) | 30 (6.4%) | 3 (8.3%) |  |
| Current smoker | 150 (1.3%) | 98 (1.2%) | 42 (1.5%) | 10 (2.1%) | 0 (0.0%) |  |
| AMH, ng/ml, median (IQR) | 4.3 (2.3, 7.4) | 4.3 (2.3, 7.5) | 4.3 (2.4, 7.3) | 4.1 (2.1, 7.0) | 3.5 (2.1, 6.3) | 0.511 |
| FSH, mIU/mL, median (IQR) | 5.8 (4.9, 6.8) | 5.8 (4.9, 6.8) | 5.8 (5.0, 6.8) | 5.7 (4.9, 6.7) | 6.1 (4.9, 7.1) | 0.512 |
| AFC, median (IQR) | 19.0 (12.0, 30.0) | 19.0 (12.0, 30.0) | 18.0 (12.0, 30.0) | 18.0 (11.0, 29.0) | 13.0 (9.0, 25.0) | 0.037 |
| ART type |  |  |  |  |  | 0.084 |
| IVF | 7,508 (66.4%) | 5,356 (66.6%) | 1,806 (65.6%) | 326 (69.4%) | 20 (55.6%) |  |
| ICSI | 2,199 (19.5%) | 1,572 (19.5%) | 547 (19.9%) | 75 (16.0%) | 5 (13.9%) |  |
| IVF+ICSI | 765 (6.8%) | 538 (6.7%) | 194 (7.1%) | 29 (6.2%) | 4 (11.1%) |  |
| PGT | 828 (7.3%) | 577 (7.2%) | 204 (7.4%) | 40 (8.5%) | 7 (19.4%) |  |
| Ovarian stimulation protocol |  |  |  |  |  | 0.051 |
| GnRH agonist | 8,841 (78.2%) | 6,306 (78.4%) | 2,161 (78.6%) | 350 (74.5%) | 24 (66.7%) |  |
| GnRH antagonist | 1,521 (13.5%) | 1,075 (13.4%) | 374 (13.6%) | 67 (14.3%) | 5 (13.9%) |  |
| Others | 938 (8.3%) | 662 (8.2%) | 216 (7.9%) | 53 (11.3%) | 7 (19.4%) |  |
| Infertility type |  |  |  |  |  | 0.338 |
| Primary infertility | 4,547 (40.2%) | 3,207 (39.9%) | 1,131 (41.1%) | 198 (42.1%) | 11 (30.6%) |  |
| Secondary infertility | 6,753 (59.8%) | 4,836 (60.1%) | 1,620 (58.9%) | 272 (57.9%) | 25 (69.4%) |  |
| Usable embryo rate, median (IQR) | 0.8 (0.6, 1.0) | 0.8 (0.6, 1.0) | 0.8 (0.6, 1.0) | 0.8 (0.6, 0.9) | 0.8 (0.6, 1.0) | 0.127 |
| Slow-cleaving embryo rate, median (IQR) | 0.5 (0.2, 0.8) | 0.5 (0.2, 0.8) | 0.5 (0.2, 0.8) | 0.5 (0.2, 0.8) | 0.5 (0.3, 0.9) | 0.745 |
| High-fragmentation embryo rate, median (IQR) | 0.0 (0.0, 0.3) | 0.0 (0.0, 0.3) | 0.0 (0.0, 0.3) | 0.0 (0.0, 0.3) | 0.0 (0.0, 0.3) | 0.525 |
| 1PN rate, median (IQR) | 0.0 (0.0, 0.1) | 0.0 (0.0, 0.1) | 0.0 (0.0, 0.1) | 0.0 (0.0, 0.1) | 0.0 (0.0, 0.0) | 0.296 |

Note: Participants were divided into four groups according to the Pittsburgh Sleep Quality Index (PSQI) score (range: 0–21): good group (0–5), fair group (6–10), poor group (11–15), and very poor group (16–21). Slight missing data existed in individual variables. All analyses were performed using valid observed data.

Abbreviations: IQR=interquartile range; BMI=body mass index; AMH=anti-Müllerian hormone; FSH=follicle stimulating hormone; AFC=antral follicle count; ART=assisted reproductive technology; IVF=in vitro fertilization; ICSI=intracytoplasmic sperm injection; GnRH=gonadotropin-releasing hormone; PGT=preimplantation genetic testing.

Supplemental table 2. **Sensitivity analysis of the relationship between sleep characteristics and cumulative live birth in 12,158 oocyte retrieval cycles.**

|  | **Cumulative live birth (1-year)** | | **Cumulative live birth (2-year)** | |
| --- | --- | --- | --- | --- |
|  | **Adjusted RR (95% CI)** | **P** | **Adjusted RR (95% CI)** | **P** |
| Sleep quality |  |  |  |  |
| Good | ref |  | ref |  |
| Fair | 0.999 (0.948-1.052) | 0.966 | 0.999 (0.951-1.051) | 0.983 |
| Poor | 0.985 (0.935-1.038) | 0.572 | 0.994 (0.945-1.045) | 0.804 |
| Very poor | **0.931 (0.883-0.982)** | **0.008*** | 1.012 (0.963-1.064) | 0.642 |
| Subjective sleep quality |  |  |  |  |
| Very good | ref |  | ref |  |
| Fairly good | 1.002 (0.959-1.048) | 0.920 | 0.998 (0.956-1.042) | 0.924 |
| Fairly bad | 1.021 (0.976-1.067) | 0.367 | 1.018 (0.975-1.062) | 0.425 |
| Very bad | 0.996 (0.953-1.041) | 0.863 | 0.99 (0.949-1.034) | 0.656 |
| Sleep latency (score) |  |  |  |  |
| 0 | ref |  | ref |  |
| 1 | 0.967 (0.926-1.011) | 0.139 | 0.965 (0.925-1.006) | 0.094 |
| 2 | 0.985 (0.943-1.029) | 0.498 | 0.983 (0.942-1.025) | 0.421 |
| 3 | 0.981 (0.939-1.025) | 0.388 | 0.971 (0.931-1.013) | 0.175 |
| Sleep duration (h) |  |  |  |  |
| >7 | ref |  | ref |  |
| 6 – 7 | 1.019 (0.963-1.077) | 0.520 | 1.029 (0.975-1.086) | 0.303 |
| 5 – <6 | **0.906 (0.855-0.96)** | **0.001*** | **0.928 (0.878-0.981)** | **0.008*** |
| <5 | 1.018 (0.962-1.076) | 0.538 | 1.051 (0.996-1.109) | 0.067 |
| Habitual sleep efficiency (%) |  |  |  |  |
| ≥85 | ref |  | ref |  |
| 75 – <85 | 1.006 (0.96-1.055) | 0.796 | 1.006 (0.961-1.053) | 0.808 |
| 65 – < 75 | 1.006 (0.96-1.055) | 0.788 | 1.005 (0.96-1.052) | 0.829 |
| < 65 | **0.928 (0.884-0.974)** | **0.002*** | **0.936 (0.893-0.98)** | **0.005*** |
| Sleep disturbances (score) |  |  |  |  |
| 0 | ref |  | ref |  |
| 1 | 1.003 (0.959-1.05) | 0.882 | 1.005 (0.962-1.05) | 0.829 |
| 2 | 0.971 (0.928-1.016) | 0.199 | 0.972 (0.93-1.015) | 0.199 |
| 3 | **1.153 (1.104-1.204)** | **<0.001*** | **1.12 (1.073-1.169)** | **<0.001*** |
| Use of sleeping medication (times/week) |  |  |  |  |
| 0 | ref |  | ref |  |
| <1 | 0.983 (0.938-1.03) | 0.471 | 0.985 (0.942-1.03) | 0.512 |
| 1 – 2 | 1.005 (0.96-1.053) | 0.824 | 1.012 (0.968-1.058) | 0.591 |
| ≥3 | 0.982 (0.937-1.029) | 0.442 | 0.987 (0.944-1.032) | 0.571 |
| Daytime dysfunction (score) |  |  |  |  |
| 0 | ref |  | ref |  |
| 1 | 1.047 (0.995-1.102) | 0.077 | 1.045 (0.995-1.098) | 0.079 |
| 2 | **0.942 (0.894-0.992)** | **0.024*** | 0.97 (0.923-1.02) | 0.236 |
| 3 | **0.752 (0.711-0.795)** | **<0.001*** | **0.787 (0.746-0.829)** | **<0.001*** |

Note: Overlapping weighted quasi-Poisson regression was used to estimate the risk ratios (RR). Adjusted factors included female age, body mass index, type of infertility, polycystic ovary syndrome, severe oligoasthenoteratozoospermia, moderate to severe intrauterine adhesions, recurrent implantation failure, recurrent miscarriage, untreated hydrosalpinx, ovarian stimulation protocol, and assisted reproductive technology type.

Supplemental table 3. **Mediating effect of available embryo rate between sleep characteristics and cumulative live birth in 10,176 oocyte retrieval cycles.**

| **Variable** | **Total effect** | | **Indirect effect** | | **Direct effect** | | **Proportion**  **Mediated(%)** |
| --- | --- | --- | --- | --- | --- | --- | --- |
|  | **Coefficients (95%CI)** | **P** | **Coefficients (95%CI)** | **P** | **Coefficients (95%CI)** | **P** |  |
| Cumulative Live Birth (1-year) |  |  |  |  |  |  |  |
| Sleep quality |  |  |  |  |  |  |  |
| Good | ref |  | ref |  | ref |  |  |
| Fair | 0.983 (0.918–1.039) | 0.591 | 1.000 (0.997–1.002) | 0.977 | 0.983 (0.918–1.039) | 0.585 | 0.20% |
| Poor | 0.995 (0.879–1.118) | 0.985 | 1.003 (0.993–1.013) | 0.5 | 0.992 (0.874–1.116) | 0.965 | -57.30% |
| Very poor | 1.030 (0.660–1.409) | 0.893 | 1.001 (0.983–1.019) | 0.989 | 1.029 (0.660–1.406) | 0.889 | 3.80% |
| Subjective sleep quality |  |  |  |  |  |  |  |
| Very good | ref |  | ref |  | ref |  |  |
| Fairly good | 1.004 (0.966–1.040) | 0.825 | 1.001 (1.000–1.002) | 0.172 | 1.004 (0.965–1.040) | 0.851 | 18.50% |
| Fairly bad | 1.022 (0.975–1.067) | 0.356 | 1.000 (0.999–1.002) | 0.823 | 1.022 (0.975–1.067) | 0.362 | 0.80% |
| Very bad | 0.989 (0.927–1.058) | 0.765 | 1.001 (0.999–1.003) | 0.467 | 0.989 (0.926–1.057) | 0.755 | -7.20% |
| Sleep latency(score) |  |  |  |  |  |  |  |
| 0 | ref |  | ref |  | ref |  |  |
| 1 | **0.965 (0.932–0.998)** | **0.040*** | 1.000 (0.999–1.001) | 0.797 | **0.966 (0.932–0.998)** | **0.040*** | **0.20%** |
| 2 | 0.980 (0.932–1.027) | 0.362 | 1.000 (0.999–1.001) | 0.949 | 0.980 (0.933–1.026) | 0.354 | 0.10% |
| 3 | 0.980 (0.896–1.065) | 0.687 | 1.001 (0.998–1.004) | 0.667 | 0.980 (0.895–1.065) | 0.679 | -3.10% |
| Sleep duration(h) |  |  |  |  |  |  |  |
| >7 | ref |  | ref |  | ref |  |  |
| 6 – 7 | 1.033 (0.964–1.107) | 0.352 | 1.000 (0.996–1.004) | 0.997 | 1.034 (0.964–1.107) | 0.358 | -0.60% |
| 5 – <6 | 0.844 (0.588–1.267) | 0.532 | 0.999 (0.970–1.023) | 0.935 | 0.844 (0.589–1.275) | 0.534 | 0.60% |
| <5 | 1.042 (0.578–1.739) | 0.819 | 0.996 (0.930–1.089) | 0.977 | 1.046 (0.574–1.729) | 0.827 | -10.30% |
| Habitual sleep efficiency(%) |  |  |  |  |  |  |  |
| ≥85 | ref |  | ref |  | ref |  |  |
| 75 – <85 | 1.006 (0.969–1.052) | 0.679 | 1.000 (0.998–1.002) | 0.829 | 1.007 (0.969–1.052) | 0.677 | -1.90% |
| 65 – < 75 | 0.995 (0.915–1.090) | 0.913 | 1.000 (0.998–1.006) | 0.813 | 0.995 (0.915–1.090) | 0.905 | -6.40% |
| < 65 | 0.903 (0.731–1.034) | 0.132 | 1.001 (0.995–1.015) | 0.675 | 0.902 (0.727–1.033) | 0.13 | -1.00% |
| Sleep disturbances(score) |  |  |  |  |  |  |  |
| 0 | ref |  | ref |  | ref |  |  |
| 1 | 1.039 (0.945–1.135) | 0.647 | 1.000 (0.995–1.003) | 0.953 | 1.039 (0.945–1.137) | 0.641 | 0.00% |
| 2 | 1.025 (0.812–1.239) | 0.829 | 1.000 (0.987–1.007) | 0.973 | 1.025 (0.814–1.236) | 0.821 | 0.40% |
| 3 | 0.869 (0.349–1.332) | 0.669 | 0.998 (0.881–1.045) | 0.849 | 0.870 (0.340–1.397) | 0.703 | 1.10% |
| Use of sleeping medication(times/week) |  |  |  |  |  |  |  |
| 0 | ref |  | ref |  | ref |  |  |
| <1 | 0.991 (0.950–1.046) | 0.751 | 1.000 (0.997–1.003) | 0.945 | 0.991 (0.950–1.046) | 0.747 | 0.50% |
| 1 – 2 | 1.011 (0.891–1.063) | 0.835 | 1.001 (0.997–1.010) | 0.396 | 1.010 (0.888–1.062) | 0.873 | 7.90% |
| ≥3 | 1.025 (0.710–1.111) | 0.683 | 1.000 (0.983–1.021) | 0.949 | 1.025 (0.722–1.115) | 0.671 | 0.50% |
| Daytime dysfunction(score) |  |  |  |  |  |  |  |
| 0 | ref |  | ref |  | ref |  |  |
| 1 | 1.017 (0.939–1.080) | 0.701 | 1.000 (0.997–1.006) | 0.955 | 1.017 (0.941–1.079) | 0.709 | 0.50% |
| 2 | 0.938 (0.844–1.061) | 0.376 | 1.001 (0.986–1.009) | 0.813 | 0.938 (0.843–1.060) | 0.376 | -0.80% |
| 3 | 0.871 (0.546–1.305) | 0.542 | 1.004 (0.908–1.031) | 0.969 | 0.867 (0.550–1.295) | 0.551 | -2.90% |
| Cumulative Live Birth (2-year) |  |  |  |  |  |  |  |
| Sleep quality |  |  |  |  |  |  |  |
| Good | ref |  | ref |  | ref |  |  |
| Fair | 0.979 (0.915–1.036) | 0.516 | 1.000 (0.997–1.003) | 0.953 | 0.979 (0.916–1.035) | 0.516 | 0.30% |
| Poor | 1.011 (0.894–1.124) | 0.827 | 1.005 (0.998–1.015) | 0.208 | 1.006 (0.889–1.119) | 0.895 | 45.60% |
| Very poor | 1.069 (0.715–1.423) | 0.759 | 1.002 (0.983–1.025) | 0.899 | 1.067 (0.714–1.423) | 0.765 | 3.00% |
| Subjective sleep quality |  |  |  |  |  |  |  |
| Very good | ref |  | ref |  | ref |  |  |
| Fairly good | 1.002 (0.967–1.035) | 0.931 | 1.001 (1.000–1.002) | 0.16 | 1.001 (0.966–1.034) | 0.953 | 47.90% |
| Fairly bad | 1.017 (0.974–1.061) | 0.444 | 1.000 (0.999–1.002) | 0.823 | 1.016 (0.974–1.061) | 0.440 | 1.10% |
| Very bad | 0.982 (0.918–1.049) | 0.613 | 1.001 (0.999–1.003) | 0.448 | 0.981 (0.918–1.049) | 0.599 | -4.50% |
| Sleep latency (score) |  |  |  |  |  |  |  |
| **Variable** | **Total effect** | | **Indirect effect** | | **Direct effect** | | **Proportion**  **Mediated(%)** |
|  | **Coefficients (95%CI)** | **P** | **Coefficients (95%CI)** | **P** | **Coefficients (95%CI)** | **P** |  |
| 0 | ref |  | ref |  | ref |  |  |
| 1 | **0.963 (0.932–0.996)*** | **0.026** | 1.000 (0.999–1.001) | 0.789 | **0.963 (0.932–0.996)*** | **0.026** | 0.20% |
| 2 | 0.976 (0.929–1.024) | 0.280 | 1.000 (0.999–1.001) | 0.947 | 0.976 (0.929–1.024) | 0.278 | 0.10% |
| 3 | 0.973 (0.891–1.059) | 0.557 | 1.001 (0.998–1.003) | 0.653 | 0.973 (0.891–1.058) | 0.546 | -2.30% |
| Sleep duration (h) |  |  |  |  |  |  |  |
| >7 | ref |  | ref |  | ref |  |  |
| 6 – 7 | 1.037 (0.968–1.103) | 0.308 | 1.000 (0.997–1.005) | 0.773 | 1.036 (0.968–1.103) | 0.308 | 1.30% |
| 5 – <6 | 0.839 (0.602–1.239) | 0.526 | 1.003 (0.983–1.032) | 0.809 | 0.837 (0.597–1.234) | 0.538 | -1.60% |
| <5 | 1.145 (0.670–1.829) | 0.611 | 1.011 (0.957–1.099) | 0.623 | 1.132 (0.666–1.795) | 0.653 | 8.00% |
| Habitual sleep efficiency (%) |  |  |  |  |  |  |  |
| ≥85 | ref |  | ref |  | ref |  |  |
| 75 – <85 | 1.004 (0.967–1.056) | 0.755 | 1.000 (0.997–1.001) | 0.753 | 1.005 (0.967–1.056) | 0.733 | -4.10% |
| 65 – < 75 | 0.994 (0.914–1.090) | 0.901 | 1.000 (0.997–1.004) | 0.761 | 0.993 (0.913–1.091) | 0.885 | -7.10% |
| < 65 | 0.924 (0.650–1.057) | 0.244 | 1.001 (0.943–1.009) | 0.593 | 0.923 (0.686–1.067) | 0.242 | -1.80% |
| Sleep disturbances (score) |  |  |  |  |  |  |  |
| 0 | ref |  | ref |  | ref |  |  |
| 1 | 1.034 (0.941–1.125) | 0.753 | 1.000 (0.997–1.004) | 0.969 | 1.034 (0.941–1.127) | 0.763 | 0.00% |
| 2 | 1.011 (0.820–1.196) | 0.841 | 1.000 (0.993–1.010) | 0.869 | 1.011 (0.823–1.192) | 0.841 | 1.60% |
| 3 | 0.840 (0.349–1.254) | 0.673 | 0.997 (0.935–1.036) | 0.835 | 0.842 (0.350–1.298) | 0.713 | 1.50% |
| Use of sleeping medication (times/week) |  |  |  |  |  |  |  |
| 0 | ref |  | ref |  | ref |  |  |
| <1 | 0.991 (0.954–1.049) | 0.767 | 1.000 (0.997–1.005) | 0.953 | 0.991 (0.954–1.046) | 0.777 | 0.50% |
| 1 – 2 | 1.017 (0.887–1.067) | 0.781 | 1.001 (0.990–1.007) | 0.451 | 1.016 (0.891–1.065) | 0.793 | 5.70% |
| ≥3 | 1.023 (0.640–1.124) | 0.827 | 1.000 (0.923–1.025) | 0.949 | 1.022 (0.659–1.137) | 0.817 | 0.60% |
| Daytime dysfunction (score) |  |  |  |  |  |  |  |
| 0 | ref |  | ref |  | ref |  |  |
| 1 | 1.016 (0.931–1.072) | 0.837 | 1.000 (0.995–1.003) | 0.979 | 1.016 (0.932–1.072) | 0.833 | 0.70% |
| 2 | 0.962 (0.867–1.089) | 0.629 | 1.001 (0.990–1.011) | 0.775 | 0.961 (0.868–1.085) | 0.615 | -1.60% |

Note: RR in this table represents the incidence rate ratio (IRR). The 95% confidence intervals were constructed using 1000 bootstrap resamples, and all models were adjusted for relevant confounding factors. Adjusted factors included female age, body mass index, type of infertility, polycystic ovary syndrome, severe oligoasthenoteratozoospermia, moderate to severe intrauterine adhesions, recurrent implantation failure, recurrent miscarriage, untreated hydrosalpinx, ovarian stimulation protocol, and assisted reproductive technology type. Mediated proportions outside the range of 0%–100% should be interpreted cautiously because ratio-based estimates may become unstable when the total effect is close to the null or when direct and indirect effects operate in opposite directions.

Supplemental table 4. **Mediating effect of slow-cleaving embryo rate between sleep characteristics and cumulative live birth in 10,176 oocyte retrieval cycles.**

| **Variable** | **Total effect** | | **Indirect effect** | | **Direct effect** | | **Proportion**  **Mediated(%)** |
| --- | --- | --- | --- | --- | --- | --- | --- |
|  | **Coefficients (95%CI)** | **P** | **Coefficients (95%CI)** | **P** | **Coefficients (95%CI)** | **P** |  |
| Cumulative Live Birth (1-year) |  |  |  |  |  |  |  |
| Sleep quality |  |  |  |  |  |  |  |
| Good | ref |  | ref |  | ref |  |  |
| Fair | 0.984 (0.920–1.041) | 0.613 | 0.996 (0.987–1.004) | 0.298 | 0.988 (0.922–1.044) | 0.697 | 23.20% |
| Poor | 0.993 (0.877–1.121) | 0.991 | 0.992 (0.969–1.010) | 0.378 | 1.002 (0.885–1.127) | 0.899 | 123.60% |
| Very poor | 1.053 (0.687–1.447) | 0.821 | 1.008 (0.945–1.077) | 0.805 | 1.044 (0.678–1.437) | 0.849 | 16.00% |
| Subjective sleep quality |  |  |  |  |  |  |  |
| Very good | ref |  | ref |  | ref |  |  |
| Fairly good | 1.005 (0.966–1.039) | 0.817 | 0.998 (0.991–1.006) | 0.657 | 1.006 (0.969–1.041) | 0.735 | -41.80% |
| Fairly bad | 1.023 (0.977–1.069) | 0.326 | 1.000 (0.990–1.010) | 0.983 | 1.023 (0.978–1.069) | 0.322 | -0.40% |
| Very bad | 0.987 (0.924–1.058) | 0.717 | **0.981 (0.964–0.998)** | **0.020*** | 1.007 (0.941–1.077) | 0.813 | 153.20% |
| Sleep latency (score) |  |  |  |  |  |  |  |
| 0 | ref |  | ref |  | ref |  |  |
| 1 | **0.965 (0.931–0.998)** | **0.038*** | 0.992 (0.984–1.000) | 0.066 | 0.973 (0.938–1.007) | 0.102 | 22.50% |
| 2 | 0.981 (0.933–1.027) | 0.362 | 0.991 (0.980–1.001) | 0.084 | 0.990 (0.941–1.037) | 0.637 | 47.70% |
| 3 | 0.979 (0.893–1.065) | 0.657 | 0.992 (0.971–1.013) | 0.453 | 0.986 (0.907–1.072) | 0.775 | 35.40% |
| Sleep duration (h) |  |  |  |  |  |  |  |
| >7 | ref |  | ref |  | ref |  |  |
| 6 – 7 | 1.033 (0.966–1.104) | 0.350 | 1.003 (0.996–1.012) | 0.436 | 1.030 (0.963–1.100) | 0.380 | 8.90% |
| 5 – <6 | 0.857 (0.605–1.275) | 0.593 | 0.986 (0.947–1.018) | 0.428 | 0.869 (0.614–1.296) | 0.639 | 9.10% |
| <5 | 1.037 (0.583–1.740) | 0.861 | 1.060 (0.995–1.158) | 0.122 | 0.978 (0.543–1.642) | 0.995 | 160.90% |
| Habitual sleep efficiency (%) |  |  |  |  |  |  |  |
| ≥85 |  |  |  |  |  |  |  |
| 75 – <85 | 1.006 (0.965–1.054) | 0.679 | 1.001 (0.992–1.010) | 0.825 | 1.005 (0.966–1.052) | 0.693 | 10.40% |
| 65 – < 75 | 0.997 (0.916–1.096) | 0.955 | 0.999 (0.981–1.016) | 0.903 | 0.998 (0.918–1.097) | 0.937 | 42.40% |
| < 65 | 0.908 (0.709–1.040) | 0.152 | 1.011 (0.938–1.035) | 0.504 | 0.898 (0.723–1.038) | 0.13 | -10.80% |
| Sleep disturbances (score) |  |  |  |  |  |  |  |
| 0 | ref |  | ref |  | ref |  |  |
| 1 | 1.034 (0.946–1.136) | 0.645 | 1.002 (0.992–1.012) | 0.665 | 1.032 (0.943–1.135) | 0.699 | 6.70% |
| 2 | 1.002 (0.784–1.207) | 0.929 | 0.985 (0.960–1.011) | 0.486 | 1.017 (0.799–1.241) | 0.881 | -856.30% |
| 3 | 0.865 (0.347–1.357) | 0.691 | 0.920 (0.788–1.060) | 0.342 | 0.940 (0.400–1.484) | 0.885 | 57.40% |
| Use of sleeping medication (times/week) |  |  |  |  |  |  |  |
| 0 | ref |  | ref |  | ref |  |  |
| <1 | 0.992 (0.952–1.049) | 0.799 | 1.002 (0.994–1.009) | 0.645 | 0.990 (0.950–1.047) | 0.743 | -31.80% |
| 1 – 2 | 1.013 (0.887–1.065) | 0.805 | 1.006 (0.984–1.016) | 0.442 | 1.007 (0.888–1.059) | 0.939 | 46.90% |
| ≥3 | 1.022 (0.691–1.125) | 0.757 | 0.990 (0.922–1.018) | 0.38 | 1.032 (0.705–1.143) | 0.599 | -44.30% |
| Daytime dysfunction (score) |  |  |  |  |  |  |  |
| 0 | ref |  | ref |  | ref |  |  |
| 1 | 1.023 (0.938–1.085) | 0.651 | 1.000 (0.990–1.010) | 0.961 | 1.022 (0.939–1.087) | 0.669 | 1.70% |
| 2 | 0.945 (0.856–1.067) | 0.434 | 0.991 (0.974–1.010) | 0.494 | 0.954 (0.863–1.071) | 0.494 | 16.00% |
| 3 | 0.882 (0.557–1.311) | 0.605 | 0.945 (0.850–1.040) | 0.49 | 0.933 (0.610–1.330) | 0.737 | 44.80% |
| Cumulative Live Birth (2-year) |  |  |  |  |  |  |  |
| Sleep quality |  |  |  |  |  |  |  |
| Good | ref |  | ref |  | ref |  |  |
| Fair | 0.980 (0.918–1.035) | 0.510 | 0.996 (0.986–1.004) | 0.306 | 0.984 (0.922–1.041) | 0.597 | 21.10% |
| Poor | 1.009 (0.899–1.117) | 0.823 | 0.991 (0.966–1.011) | 0.376 | 1.018 (0.904–1.131) | 0.731 | -108.30% |
| Very poor | 1.091 (0.723–1.461) | 0.683 | 1.009 (0.942–1.084) | 0.811 | 1.081 (0.733–1.451) | 0.697 | 10.50% |
| Subjective sleep quality |  |  |  |  |  |  |  |
| Very good | ref |  | ref |  | ref |  |  |
| Fairly good | 1.002 (0.965–1.036) | 0.927 | 0.998 (0.990–1.006) | 0.651 | 1.004 (0.967–1.036) | 0.831 | -98.90% |
| Fairly bad | 1.018 (0.974–1.063) | 0.402 | 1.000 (0.990–1.010) | 0.979 | 1.018 (0.973–1.061) | 0.376 | -0.50% |
| Very bad | 0.980 (0.915–1.049) | 0.555 | **0.980 (0.963–0.998)** | **0.022*** | 1.000 (0.937–1.064) | 0.971 | 99.50% |
| Sleep latency (score) |  |  |  |  |  |  |  |
| **Variable** | **Total effect** | | **Indirect effect** | | **Direct effect** | | **Proportion**  **Mediated(%)** |
|  | **Coefficients (95%CI)** | **P** | **Coefficients (95%CI)** | **P** | **Coefficients (95%CI)** | **P** |  |
| 0 | ref |  | ref |  | ref |  |  |
| 1 | **0.963 (0.931–0.996)** | **0.028*** | 0.992 (0.984–1.001) | 0.072 | 0.971 (0.938–1.003) | 0.082 | 21.50% |
| 2 | 0.977 (0.930–1.025) | 0.286 | 0.991 (0.979–1.002) | 0.092 | 0.986 (0.937–1.031) | 0.500 | 40.40% |
| 3 | 0.972 (0.891–1.057) | 0.546 | 0.992 (0.970–1.014) | 0.453 | 0.979 (0.902–1.065) | 0.661 | 27.40% |
| Sleep duration(h) |  |  |  |  |  |  |  |
| >7 | ref |  | ref |  | ref |  |  |
| 6 – 7 | 1.036 (0.969–1.104) | 0.298 | 1.004 (0.995–1.014) | 0.448 | 1.032 (0.967–1.098) | 0.336 | 10.20% |
| 5 – <6 | 0.855 (0.613–1.247) | 0.593 | 0.983 (0.938–1.022) | 0.434 | 0.870 (0.626–1.266) | 0.659 | 11.20% |
| <5 | 1.140 (0.665–1.852) | 0.635 | 1.075 (0.996–1.179) | 0.112 | 1.060 (0.622–1.667) | 0.783 | 55.60% |
| Habitual sleep efficiency(%) |  |  |  |  |  |  |  |
| ≥85 | ref |  | ref |  | ref |  |  |
| 75 – <85 | 1.004 (0.966–1.057) | 0.759 | 1.001 (0.991–1.010) | 0.835 | 1.003 (0.966–1.058) | 0.805 | 16.20% |
| 65 – < 75 | 0.996 (0.913–1.096) | 0.939 | 0.999 (0.980–1.016) | 0.893 | 0.997 (0.916–1.099) | 0.957 | 29.20% |
| < 65 | 0.929 (0.659–1.065) | 0.262 | 1.011 (0.905–1.036) | 0.530 | 0.919 (0.690–1.060) | 0.204 | -14.70% |
| Sleep disturbances(score) |  |  |  |  |  |  |  |
| 0 | ref |  | ref |  | ref |  |  |
| 1 | 1.029 (0.943–1.127) | 0.777 | 1.002 (0.993–1.012) | 0.671 | 1.027 (0.941–1.121) | 0.821 | 7.90% |
| 2 | 0.988 (0.810–1.173) | 0.941 | 0.985 (0.960–1.013) | 0.565 | 1.003 (0.821–1.191) | 0.867 | 124.00% |
| 3 | 0.835 (0.349–1.267) | 0.683 | 0.920 (0.786–1.052) | 0.328 | 0.908 (0.405–1.409) | 0.877 | 46.30% |
| Use of sleeping medication(times/week) |  |  |  |  |  |  |  |
| 0 | ref |  | ref |  | ref |  |  |
| <1 | 0.993 (0.956–1.052) | 0.829 | 1.003 (0.994–1.010) | 0.629 | 0.990 (0.955–1.051) | 0.765 | -34.40% |
| 1 – 2 | 1.019 (0.894–1.069) | 0.735 | 1.007 (0.983–1.017) | 0.520 | 1.013 (0.896–1.064) | 0.853 | 34.10% |
| ≥3 | 1.019 (0.608–1.114) | 0.885 | 0.990 (0.902–1.025) | 0.396 | 1.030 (0.642–1.134) | 0.725 | -52.60% |
| Daytime dysfunction(score) |  |  |  |  |  |  |  |
| 0 | ref |  | ref |  | ref |  |  |
| 1 | 1.021 (0.932–1.078) | 0.745 | 1.000 (0.990–1.010) | 0.907 | 1.021 (0.933–1.077) | 0.737 | 1.90% |
| 2 | 0.969 (0.878–1.090) | 0.729 | 0.991 (0.974–1.011) | 0.520 | 0.979 (0.890–1.093) | 0.817 | 30.30% |
| 3 | 0.855 (0.556–1.360) | 0.629 | 0.943 (0.847–1.048) | 0.546 | 0.907 (0.614–1.356) | 0.735 | 37.50% |

Note: RR in this table represents the incidence rate ratio (IRR). The 95% confidence intervals were constructed using 1000 bootstrap resamples, and all models were adjusted for relevant confounding factors. Adjusted factors included female age, body mass index, type of infertility, polycystic ovary syndrome, severe oligoasthenoteratozoospermia, moderate to severe intrauterine adhesions, recurrent implantation failure, recurrent miscarriage, untreated hydrosalpinx, ovarian stimulation protocol, and assisted reproductive technology type. Mediated proportions outside the range of 0%–100% should be interpreted cautiously because ratio-based estimates may become unstable when the total effect is close to the null or when direct and indirect effects operate in opposite directions.

Supplemental table 5. **Mediating effect of high fragmentation rate between sleep characteristics and cumulative live birth in 10,176 oocyte retrieval cycles**

| **Variable** | **Total effect** | | **Indirect effect** | | **Direct effect** | | **Proportion**  **Mediated(%)** |
| --- | --- | --- | --- | --- | --- | --- | --- |
|  | **Coefficients (95%CI)** | **P** | **Coefficients (95%CI)** | **P** | **Coefficients (95%CI)** | **P** |  |
| Cumulative Live Birth (1-year) |  |  |  |  |  |  |  |
| Sleep quality |  |  |  |  |  |  |  |
| Good | ref |  | ref |  | ref |  |  |
| Fair | 0.985 (0.919–1.041) | 0.627 | 0.996 (0.989–1.001) | 0.098 | 0.988 (0.925–1.047) | 0.717 | 24.90% |
| Poor | 0.996 (0.879–1.120) | 0.977 | 1.001 (0.990–1.012) | 0.935 | 0.995 (0.881–1.119) | 0.997 | -16.10% |
| Very poor | 1.015 (0.655–1.378) | 0.945 | 1.017 (0.987–1.053) | 0.254 | 0.998 (0.651–1.366) | 0.999 | 115.70% |
| Subjective sleep quality |  |  |  |  |  |  |  |
| Very good | ref |  | ref |  | ref |  |  |
| Fairly good | 1.005 (0.966–1.040) | 0.787 | 0.999 (0.993–1.005) | 0.809 | 1.006 (0.967–1.041) | 0.771 | -12.30% |
| Fairly bad | 1.026 (0.978–1.073) | 0.292 | 1.001 (0.993–1.009) | 0.835 | 1.025 (0.978–1.070) | 0.296 | 3.80% |
| Very bad | 0.986 (0.923–1.056) | 0.681 | **0.985 (0.972–0.998)** | **0.024*** | 1.000 (0.937–1.070) | 0.953 | 103.20% |
| Sleep latency (score) |  |  |  |  |  |  |  |
| 0 | ref |  | ref |  | ref |  |  |
| 1 | 0.967 (0.933–1.000) | 0.050 | **0.994 (0.988–1.000)** | **0.044*** | 0.973 (0.939–1.006) | 0.106 | 18.60% |
| 2 | 0.982 (0.934–1.030) | 0.426 | 0.993 (0.984–1.001) | 0.074 | 0.990 (0.942–1.035) | 0.619 | 41.10% |
| 3 | 0.983 (0.899–1.068) | 0.745 | 0.996 (0.979–1.013) | 0.595 | 0.987 (0.903–1.071) | 0.803 | 24.90% |
| Sleep duration (h) |  |  |  |  |  |  |  |
| >7 | ref |  | ref |  | ref |  |  |
| 6 – 7 | 1.035 (0.967–1.107) | 0.330 | 1.000 (0.996–1.006) | 0.853 | 1.034 (0.966–1.107) | 0.336 | 1.10% |
| 5 – <6 | 0.848 (0.595–1.254) | 0.571 | 0.993 (0.951–1.017) | 0.548 | 0.854 (0.605–1.268) | 0.589 | 4.40% |
| <5 | 1.042 (0.579–1.726) | 0.855 | 1.017 (0.981–1.091) | 0.342 | 1.025 (0.568–1.706) | 0.905 | 40.50% |
| Habitual sleep efficiency (%) |  |  |  |  |  |  |  |
| ≥85 | ref |  | ref |  | ref |  |  |
| 75 – <85 | 1.004 (0.965–1.055) | 0.743 | 1.000 (0.993–1.006) | 0.989 | 1.004 (0.965–1.058) | 0.749 | 1.70% |
| 65 – < 75 | 0.995 (0.911–1.093) | 0.913 | 1.007 (0.990–1.019) | 0.308 | 0.988 (0.908–1.094) | 0.805 | -145.50% |
| < 65 | 0.905 (0.683–1.046) | 0.16 | 1.006 (0.912–1.028) | 0.631 | 0.900 (0.702–1.050) | 0.150 | -6.00% |
| Sleep disturbances (score) |  |  |  |  |  |  |  |
| 0 | ref |  | ref |  | ref |  |  |
| 1 | 1.039 (0.945–1.135) | 0.647 | 1.000 (0.995–1.003) | 0.953 | 1.039 (0.945–1.137) | 0.641 | 0.00% |
| 2 | 1.025 (0.812–1.239) | 0.829 | 1.000 (0.987–1.007) | 0.973 | 1.025 (0.814–1.236) | 0.821 | 0.40% |
| 3 | 0.869 (0.349–1.332) | 0.669 | 0.998 (0.881–1.045) | 0.849 | 0.870 (0.340–1.397) | 0.703 | 1.10% |
| Use of sleeping medication (times/week) |  |  |  |  |  |  |  |
| 0 | ref |  | ref |  | ref |  |  |
| <1 | 0.990 (0.950–1.048) | 0.785 | 0.997 (0.990–1.009) | 0.569 | 0.994 (0.953–1.047) | 0.865 | 35.40% |
| 1 – 2 | 1.014 (0.879–1.066) | 0.837 | 1.007 (0.983–1.014) | 0.446 | 1.007 (0.883–1.060) | 0.955 | 48.10% |
| ≥3 | 1.020 (0.649–1.126) | 0.811 | 0.995 (0.909–1.023) | 0.51 | 1.025 (0.676–1.132) | 0.699 | -27.40% |
| Daytime dysfunction (score) |  |  |  |  |  |  |  |
| 0 | ref |  | ref |  | ref |  |  |
| 1 | 1.020 (0.936–1.080) | 0.703 | 1.005 (0.992–1.013) | 0.645 | 1.015 (0.938–1.075) | 0.787 | 23.70% |
| 2 | 0.948 (0.853–1.069) | 0.467 | 0.995 (0.980–1.012) | 0.737 | 0.953 (0.857–1.074) | 0.500 | 9.30% |
| 3 | 0.871 (0.553–1.319) | 0.573 | 0.952 (0.854–1.040) | 0.559 | 0.915 (0.611–1.324) | 0.671 | 35.80% |
| Cumulative Live Birth (2-year) |  |  |  |  |  |  |  |
| Sleep quality |  |  |  |  |  |  |  |
| Good | ref |  | ref |  | ref |  |  |
| Fair | 0.981 (0.918–1.037) | 0.528 | 0.996 (0.988–1.001) | 0.104 | 0.985 (0.923–1.044) | 0.633 | 22.30% |
| Poor | 1.013 (0.898–1.127) | 0.817 | 1.001 (0.989–1.013) | 0.939 | 1.012 (0.899–1.125) | 0.823 | 5.70% |
| Very poor | 1.048 (0.705–1.406) | 0.831 | 1.019 (0.987–1.058) | 0.256 | 1.028 (0.689–1.391) | 0.905 | 40.10% |
| Subjective sleep quality |  |  |  |  |  |  |  |
| Very good | ref |  | ref |  | ref |  |  |
| Fairly good | 1.003 (0.967–1.037) | 0.905 | 0.999 (0.993–1.006) | 0.803 | 1.003 (0.966–1.036) | 0.865 | -25.40% |
| Fairly bad | 1.021 (0.977–1.065) | 0.352 | 1.001 (0.992–1.010) | 0.835 | 1.020 (0.977–1.062) | 0.342 | 4.90% |
| Very bad | 0.979 (0.916–1.045) | 0.522 | **0.985 (0.971–0.998)** | **0.032*** | 0.993 (0.930–1.059) | 0.881 | 69.20% |
| Sleep latency(score) |  |  |  |  |  |  |  |
| **Variable** | **Total effect** | | **Indirect effect** | | **Direct effect** | | **Proportion**  **Mediated(%)** |
|  | **Coefficients (95%CI)** | **P** | **Coefficients (95%CI)** | **P** | **Coefficients (95%CI)** | **P** |  |
| 0 | ref |  | ref |  | ref |  |  |
| 1 | **0.965 (0.933–0.996)** | **0.030*** | **0.994 (0.987–1.000)** | **0.048*** | 0.971 (0.939–1.002) | 0.066 | 17.70% |
| 2 | 0.979 (0.931–1.026) | 0.312 | 0.993 (0.984–1.001) | 0.078 | 0.986 (0.939–1.032) | 0.502 | 34.40% |
| 3 | 0.976 (0.896–1.062) | 0.611 | 0.996 (0.979–1.014) | 0.595 | 0.981 (0.900–1.067) | 0.673 | 18.10% |
| Sleep duration (h) |  |  |  |  |  |  |  |
| >7 | ref |  | ref |  | ref |  |  |
| 6 – 7 | 1.038 (0.970–1.107) | 0.296 | 1.001 (0.996–1.006) | 0.851 | 1.038 (0.969–1.105) | 0.298 | 1.40% |
| 5 – <6 | 0.844 (0.593–1.236) | 0.548 | 0.990 (0.944–1.015) | 0.508 | 0.852 (0.605–1.241) | 0.585 | 6.10% |
| <5 | 1.146 (0.681–1.852) | 0.605 | 1.024 (0.986–1.098) | 0.27 | 1.119 (0.650–1.783) | 0.669 | 17.40% |
| Habitual sleep efficiency (%) |  |  |  |  |  |  |  |
| ≥85 | ref |  | ref |  | ref |  |  |
| 75 – <85 | 1.002 (0.965–1.055) | 0.837 | 1.000 (0.993–1.007) | 0.997 | 1.002 (0.966–1.055) | 0.817 | 3.10% |
| 65 – < 75 | 0.994 (0.912–1.085) | 0.891 | 1.007 (0.989–1.019) | 0.322 | 0.987 (0.908–1.081) | 0.761 | -114.10% |
| < 65 | 0.927 (0.635–1.054) | 0.252 | 1.006 (0.906–1.028) | 0.653 | 0.921 (0.666–1.058) | 0.214 | -7.70% |
| Sleep disturbances (score) |  |  |  |  |  |  |  |
| 0 | ref |  | ref |  | ref |  |  |
| 1 | 1.022 (0.938–1.111) | 0.881 | 0.992 (0.981–1.005) | 0.35 | 1.030 (0.942–1.121) | 0.785 | -35.80% |
| 2 | 0.986 (0.821–1.168) | 0.967 | 0.990 (0.963–1.017) | 0.845 | 0.996 (0.832–1.172) | 0.931 | 71.30% |
| 3 | 0.799 (0.339–1.207) | 0.631 | 0.907 (0.788–1.013) | 0.250 | 0.881 (0.413–1.263) | 0.777 | 43.60% |
| Use of sleeping medication (times/week) |  |  |  |  |  |  |  |
| 0 | ref |  | ref |  | ref |  |  |
| <1 | 0.990 (0.952–1.050) | 0.773 | 0.996 (0.990–1.010) | 0.603 | 0.994 (0.955–1.046) | 0.871 | 36.90% |
| 1 – 2 | 1.020 (0.897–1.070) | 0.715 | 1.007 (0.982–1.015) | 0.476 | 1.013 (0.897–1.064) | 0.819 | 34.90% |
| ≥3 | 1.018 (0.607–1.124) | 0.925 | 0.994 (0.906–1.025) | 0.512 | 1.023 (0.631–1.121) | 0.825 | -32.30% |
| Daytime dysfunction(score) |  |  |  |  |  |  |  |
| 0 | ref |  | ref |  | ref |  |  |
| 1 | 1.018 (0.929–1.073) | 0.815 | 1.005 (0.991–1.013) | 0.705 | 1.014 (0.932–1.068) | 0.855 | 26.30% |
| 2 | 0.972 (0.879–1.091) | 0.789 | 0.995 (0.979–1.012) | 0.771 | 0.977 (0.882–1.093) | 0.833 | 18.00% |
| 3 | 0.844 (0.556–1.336) | 0.591 | 0.951 (0.852–1.045) | 0.593 | 0.888 (0.613–1.324) | 0.643 | 29.80% |

Note: RR in this table represents the incidence rate ratio (IRR). The 95% confidence intervals were constructed using 1000 bootstrap resamples, and all models were adjusted for relevant confounding factors. Adjusted factors included female age, body mass index, type of infertility, polycystic ovary syndrome, severe oligoasthenoteratozoospermia, moderate to severe intrauterine adhesions, recurrent implantation failure, recurrent miscarriage, untreated hydrosalpinx, ovarian stimulation protocol, and assisted reproductive technology type. Mediated proportions outside the range of 0%–100% should be interpreted cautiously because ratio-based estimates may become unstable when the total effect is close to the null or when direct and indirect effects operate in opposite directions.
